# Genome-wide association study identifies and validates genetic variation in the RIG-I/MAVS signaling pathway associated with HIV-related Kaposi sarcoma in children and adults

**DOI:** 10.3389/fonc.2026.1796311

**Published:** 2026-06-29

**Authors:** Casey L. McAtee, Erin Peckham-Gregory, Pagna Sok, Melissa Richard, Luis Olivares, Deborah Marquez-Do, Grace Kisitu, Jeffrey Martin, Nader Kim El-Mallawany, Carl E. Allen, Joseph Lubega, Michael E. Scheurer

**Affiliations:** 1 Department of Pediatrics, Baylor College of Medicine, Houston, TX, United States; 2 Baylor Children’s Foundation Uganda, Kampala, Uganda; 3 Department of Epidemiology and Biostatistics, University of California San Francisco, San Francisco, CA, United States

**Keywords:** HHV-8, HIV-associated cancers, KS, KSHV, LMIC, MAVS, RIG-I

## Abstract

**Introduction:**

Immune deficiency and infection with human herpesvirus-8 (HHV-8) are established component causes of Kaposi sarcoma (KS), but they are insufficient to cause disease in most people at risk. Therefore, we hypothesized that genetic variation may contribute to KS risk in children and adults, as well as contribute to its unique epidemiology in sub-Saharan Africa.

**Methods:**

To test this hypothesis, we conducted a two-phase genome-wide association study (GWAS): a discovery study in perinatally HIV-infected children (45 KS cases, 91 controls), followed by validation of significant results in a sample of adults living with HIV (215 cases, 262 controls). After standard quality control, we tested single-nucleotide variants for association with KS.

**Results:**

In children, the top signal was a missense variant in the mitochondrial antiviral-signaling (*MAVS*) gene (rs7269320), which showed a suggestive allelic association with KS (OR 3.8, 95% CI 2.2 -6.7, p=9.7×10^-7^). In the adult validation study, the same MAVS variant was again associated with KS, indicating a recessive inheritance pattern (OR 2.2, 95% CI 1.2 -3.9, p=0.006). *MAVS* is a component of the RIG-I/MAVS signaling pathway and a biologically plausible genetic variant contributing to KS oncogenesis. MAVS protein deficiency/dysfunction is known to impair the antiviral cytokine response to HHV-8 infection and enhance its reactivation.

**Discussion:**

These findings provide the first pediatric evidence of germline susceptibility to KS and suggest RIG-I/MAVS as a candidate pathway for host risk. Further replication and functional studies are needed to clarify the mechanism underlying the risk associated with *MAVS* and to assess whether it could be used to identify individuals at the highest risk for KS in high-burden settings and/or be leveraged as a novel therapeutic strategy.

## Introduction

Kaposi sarcoma (KS) is the most common HIV-related cancer in children and adults (1, 2). KS is a malignancy of lymphoid endothelial cells driven by infection with human herpesvirus 8 (HHV-8), also known as Kaposi sarcoma herpesvirus. The incidence of HIV-related KS has fallen with the success of antiretroviral programs, but it remains relatively common in regions of the world where HIV and HHV-8 are most prevalent (3–6).

The pathogenesis and epidemiology of KS remain poorly understood compared with those of cancers more common in high-income countries. Infection with HHV-8 is a necessary component cause of KS, but most people infected with HHV-8 will not develop the disease (7, 8). In populations where HHV-8 seroprevalence is high, only a minority of patients with HIV develop KS, even at the diagnosis of HIV when CD4 counts are low, and HIV viral load is high (4, 9). Furthermore, while KS most often occurs in the setting of T cell suppression, it may develop in persons living with HIV with normal CD4 counts many years after starting antiretroviral therapy (10, 11). Finally, the endemic, HIV-unassociated variant of KS found in otherwise healthy young people in Africa occurs without any recognized immune deficiency at all (12).

Besides HIV infection, there are no known population-level risk factors for developing KS among children and young adults infected with HHV-8. In this study, we aimed to identify genetic risk factors for HIV-associated KS that could be leveraged to identify those at highest risk for the disease as well as to identify novel avenues for KS-related drug discovery. We, therefore, hypothesized that genetic variation related to the immune response to HHV-8 and/or the maintenance of the tumor immune microenvironment was associated with incident KS. To test this hypothesis, we conducted a genome-wide association study (GWAS) of HIV-associated KS among Ugandan children and adults.

## Methods

### Patient population

The study was divided into two phases: a discovery GWAS using samples from Ugandan children, and a validation analysis using samples from Ugandan adults provided by the AIDS and Cancer Specimen Resource (ACSR), funded by the United States National Cancer Institute. The discovery population was a convenience sample of perinatally infected children living with HIV aged <18 years treated between 2004–2017 by the Baylor College of Medicine Children’s Foundation – Uganda. The clinic is located in Kampala and primarily serves the Central Region of Uganda. Pediatric cases were defined as children with HIV-related KS, and controls were children with HIV infection without a history of KS at the time of enrollment. Cases and controls were unmatched. HHV-8 serostatus was unavailable for pediatric control samples.

The validation population was comprised of subjects from two adult HIV studies. Cases were ART-naïve adults living with HIV aged ≥18 years with *de novo* KS enrolled on the Anti-Retrovirals for Kaposi’s Sarcoma (ARKS) trial in Kampala, Uganda (2007-2012, NCT00444379). Controls were age-matched ART-naïve adults living with HIV enrolled on the International HIV Antiretroviral Adherence, Resistance, and Survival (UARTO) trial in Kampala and Mbarara, Uganda (2004-2015, NCT01596322). Study sites in Kampala and Mbarara primarily served the Central and Western Regions of Uganda, respectively. To include only controls at risk for epidemic KS (i.e., with HIV and HHV-8 co-infection), the adult experiment was conducted utilizing only control subjects with known seropositivity to HHV-8 by ELISA (KS cases were assumed to have HHV-8 infection as it is required to develop KS).

### Blood sample processing and genotyping

Pediatric blood samples were collected during routine clinic visits, and adult samples were collected at relevant study entry (i.e., approximately the time of HIV diagnosis and ART initiation). DNA was extracted at Baylor College of Medicine (Houston, TX) from peripheral blood mononuclear cells or buffy coat using the Prepito Blood600 Kit (Chemagin, Westphalia, Germany) and was quantified with the QuBit dsDNA Broad Range assay (Invitrogen; Carlsbad, CA).

Genotyping was conducted at the Avera Institute for Human Genetics (Sioux Falls, SD). Genotyping of pediatric samples was conducted on the Illumina (San Diego, CA) Infinium Multi-Ethnic AMR/AFR-8 kit (1,430,141 probes), which is enriched for base pair loci with high minor allele frequencies in the African/African American population. Adult samples were genotyped on the Illumina Infinium Global Screening Array-24 v1.0 (618,540 probes).

### Quality control of genotyping data

Raw genotyping data were processed into MAP and PED files using GenomeStudio v2.0 (Illumina). Standard quality control (QC) procedures for GWAS data were followed (13). In summary, subjects were removed if genotyping call rate was <95%, if recorded sex data were discordant with genotyped data, if allelic heterozygosity differed by ≥3 standard deviations from the sample mean, or if identity-by-descent exceeded 0.1875. Individual single-nucleotide variant (SNV) markers were filtered for a minor allele frequency of <5%, a call failure of ≥5%, if they were outside of Hardy-Weinberg Equilibrium (p < 0.05), or if call rates diverged between cases and controls (p <0.05). For the X-chromosome analysis, subjects were stratified by sex, and the QC process was repeated in accordance with published protocols (14). An additional test excluding SNVs with differential call rates between sexes (p <0.05) was used. Principal components analysis was used to assess for population stratification (13, 16).

### Sample size and statistical analysis

A convenience sample of all pediatric samples available to the study team was used. While the sample size available for the discovery experiment was smaller than in typical GWAS, we hypothesized that effect sizes of potential SNV-phenotype associations would be relatively large in children, a common phenomenon in pediatric GWAS, thereby increasing power (15, 16).

In the discovery experiment, we performed two tests to screen for SNV associations. The first was a one degree of freedom χ^2^ allelic test for an association between the minor allele at a given locus (*a*) and the outcome (KS). The second was a log-additive logistic regression model testing the hypothesis that one copy of the minor allele at locus *X* (*Aa*) increases the odds of KS by a factor of *γ* while two copies (*aa*) increase it by 2*γ*. In the larger validation test, we included univariable logistic regression models for dominance-deviation, recessive inheritance, and dominant inheritance.

Genomic inflation for each association test was assessed by examining Q-Q plots and calculating the genomic inflation factor λ. To account for multiple testing in the discovery experiment, p-value thresholds established by the National Human Genome Research Institute were used, with p < 5×10^-8^ indicating genome-wide significance, and p < 1×10^-5^ suggesting an association (17). A lower threshold, p < 1 × 10^-3^ was used for X chromosome analysis, given the smaller number of markers tested (18). An α of 0.05 was used in the validation study for any SNV identified a priori in the discovery set.

Linkage disequilibrium of SNVs was evaluated with HaploReg v5 (Broad Institute, Cambridge, MA) (19). Functional consequences of significant SNVs were assessed using PolyPhen-2 and SIFT (20). Relevant continuous and categorical demographic data were compared between cases and controls using Welch’s t-test and Pearson’s χ^2^ tests. Associations between SNVs and KS were reported as odds ratios (ORs).

All QC and statistical analyses were conducted in R (v4.4.2) using PLINK (v1.9) and XWAS (v3) (13, 21, 22). Ethical approval for this research was obtained from Baylor College of Medicine in Houston and Makerere University in Kampala. Sample collection as a part of the original ARKS and UARTO studies were approved by each clinical site's institutional ethics committees.

## Results

Two pediatric subjects (2%) were removed during QC, yielding 136 patients (45 cases, 91 controls) in the final analysis. Nine adult subjects (1.9%) failed QC, yielding 477 patients (215 cases, 262 controls) in the final analysis. Population stratification was not significant in either the pediatric or adult populations.

Children with KS were younger than controls (mean 9.4 vs. 15.1 years; p < 0.001). There was no significant difference in age at start of antiretroviral therapy between cases and controls (mean 8.6 vs. 7.7 years; p=0.4). The most common KS phenotype in children was lymph node predominant disease (36%), followed by skin/oral-predominant disease (31%).

Characteristics of the ARKS cohort have been previously reported (23, 24). In summary, all patients were ART-naïve at enrollment, and 69% had CD4 counts <200 cells/µL. Characteristics of the UARTO cohort of Ugandan adults living with HIV have also been previously reported (25–27). Patients were ART-naïve at enrollment, with a median baseline CD4 count typically <200 cells/µL across studies. Cases and controls in this GWAS were age-matched with a median age of 34 years (IQR, 28-40).

### Discovery pediatric study

**Figure 1:**
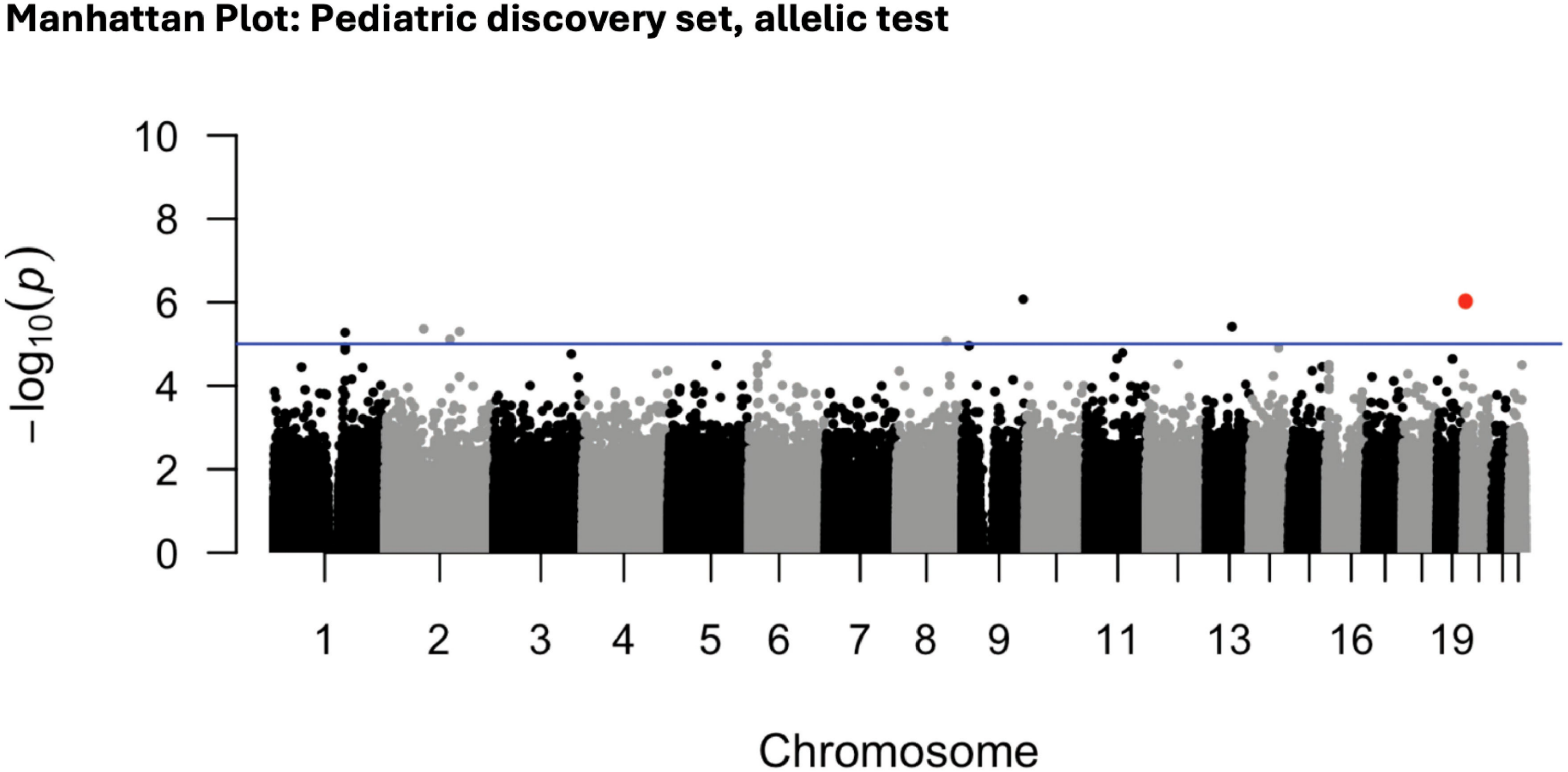
Manhattan plot: pediatric discovery set, allelic test. The rs7269320 single nucleotide variant in MAVS is marked in red. The National Human Genome Research Institute threshold for a suggested association of p < 1×10^-5^ is indicated by the blue line.

Following QC, 928,463 pediatric markers (64.9%) were analyzed. Of these, eight autosomal SNVs (**Supplementary Table**) met the National Human Genome Research Institute suggestive threshold of p < 1×10^-5^ but did not meet the more conservative genome-wide threshold of p < 5×10^-8^ (**Figure 1**). A Q-Q plot showed close agreement between observed and expected p-value distributions, with minimal genomic inflation (λ = 1.04), suggesting little evidence of residual population stratification in the pediatric allelic association analysis (**Supplementary Figure 1**). The allelic association test used in the discovery experiment was not adjusted for principal components.

The most biologically plausible finding in the discovery GWAS was an association between KS and a missense SNV located in the gene Mitochondrial Antiviral Signaling Protein (*MAVS*; rs7269320; C/T; OR, 3.8; 95% CI, 2.2-6.7; p = 9.7×10^-7^). *MAVS* is a component of the innate immune system within the RIG-I/MAVS signaling pathway, responsible for generating a cellular, cytokine-mediated antiviral response to infections, including HHV-8 infection (28, 29). The rs7269320 SNV is relatively common in African populations, with a minor allele frequency of 0.41 in the 1000 Genomes Phase 1 African population (30). Its global minor allele frequency is 0.21 (30).

A log-additive logistic regression model of the pediatric data similarly supported an association with *MAVS* (OR, 4.9; 95% CI, 2.5-9.8); p = 5.5×10^-6^. The model showed no signs of genomic inflation (λ = 0.95; **Supplementary Figure 2**). A sensitivity analysis that adjusted for a single principal component yielded findings similar to those of the unadjusted analysis (**Supplementary Analysis 1**).

The marker rs7269320 is a missense SNV resulting in an amino acid replacement of phenylalanine for serine at position 409 of the MAVS protein (31). The rs7269320 SNV is predicted to be deleterious to MAVS protein function within both SIFT (score = 0.02) and PolyPhen-2 (score = 0.99; sensitivity = 0.72; specificity = 0.97) algorithms. Within HaploReg, six other SNVs within MAVS were found to be in linkage disequilibrium with rs7269320 at *r*^2^ > 0.8, including a second missense SNV in *MAVS*, rs7262903.

The remaining seven suggested SNVs in the GWAS had no apparent biologically plausible relationship with KS or HHV-8 infection (**Supplementary Table**). No significant associations were shown on X chromosome analysis.

### Replication adult study

The rs7269320 SNV in *MAVS* was chosen for validation in an independent population. Neither the allelic nor the log-additive regression model in adults showed a significant genotype-phenotype association between *MAVS* and KS (**Tables 1**, **2**). However, the dominance-deviation logistic regression model showed deviation from the additive model towards a recessive or dominant genotype-phenotype relationship (p = 0.02).

**Table 1:** Distribution of MAVS rs7269320 minor allele and genotypes in pediatric and adult samples.

Cohort	N	Minor allele (*a)* frequency (prop)	*AA* genotype (n, prop)	*Aa* genotype (n, prop)	*aa* genotype (n, prop)
Pediatric	136				
Cases	45	0.19	11 (0.24)	25 (0.56)	9 (0.2)
Controls	91	0.48	57 (0.63)	33 (0.36)	1 (0.01)
Adult	477				
Cases	215	0.38	86 (0.4)	93 (0.43)	36 (0.17)
Controls	262	0.34	107 (0.41)	133 (0.51)	22 (0.08)

Legend: prop, proportion.

**Table 2:** Genotype-phenotype modeling of *MAVS* rs7269320 and Kaposi sarcoma.

Model	Odds ratio	95% CI	p-value
Pediatric			
Allelic	3.8	2.2, 6.7	9.7 × 10^-7^
Additive	4.9	2.5, 9.8	9.7 × 10^-6^
Dominant-deviation			0.4
Recessive	22.5	2.8, 184	0.004
Dominant	5.2	2.3, 11.6	5.8 × 10^-5^
Adult			
Allelic	1.2	0.93, 1.6	0.1
Additive	1.2	0.94, 1.6	0.1
Dominant-deviation			0.02
Recessive	2.2	1.2, 3.9	0.006
Dominant	1.0	0.7, 1.6	0.9

A recessive logistic regression model that assumes two copies of the SNV (*aa*) are required for disease showed a highly significant association between *MAVS* and KS (OR, 2.2, 95% CI 1.2-3.9; p = 0.006). A Q-Q plot showed close agreement between observed and expected p-value distributions (λ = 1.00), suggesting against population stratification in this analysis (**Supplementary Figure 3**). A sensitivity analysis adjusting for the first principal component showed no substantial change in the MAVS/KS association (**Supplementary Analysis 1**). A dominant logistic regression model did not show a significant relationship between *MAVS* and KS.

## Discussion

We conducted a first-of-its-kind GWAS to determine germline risk factors for HIV-related KS. The pediatric discovery study identified a SNV in the *MAVS gene* that was significantly associated with KS, a finding validated in an independent adult population and suggesting an autosomal recessive genotype-phenotype relationship. Given its known role in the immune response against HHV-8, the involvement of MAVS in KS oncogenesis, vis-à-vis a missense mutation, is highly plausible.

MAVS protein is involved in the innate intracellular immune response to viral infection, and it has been specifically linked to HHV-8 infection (28, 29, 32–34). The MAVS protein lies within the RIG-I intracellular signaling pathway, which detects intracellular viral RNA during active infection (28). Upon viral RNA detection, RIG-I transmits intracellular signals through mitochondrial and peroxisomal membrane-bound MAVS to produce transcription factors NF-κB, IRF3, and IRF7, ultimately producing pro-inflammatory cytokines including interferon-β, CCL-5, and IL-6, which facilitate the innate anti-viral response (28, 35). Dysfunctional or depleted MAVS is known to diminish cellular production of interferon-β production during viral infection (28, 29).

Furthermore, MAVS function is specifically related to HHV-8 infection. HHV-8 evades innate immune signaling by targeting pathways specifically involving the RIG-I/MAVS/type I interferon axis, including through altering mitochondrial dynamics to suppress host antiviral responses and promote viral production (36, 37). MAVS depletion or dysfunction in the setting of HHV-8 infection results in decreased antiviral cytokine production (28). When functional MAVS is absent, HHV-8 viral gene expression increases, latently infected cells become reactivated, and HHV-8 viral load increases (28, 29). The downstream effects of HHV-8 viral proteins on the cell cycle drive the oncogenesis of KS and other HHV-8-associated neoplasms (38).

Finally, the RIG-I/MAVS signaling pathway is involved in the development of neoplasms themselves, including KS. RIG-I deficiency was recently associated with susceptibility to classic KS in adults, implicating impaired upstream viral sensing as a potential inborn error of immunity predisposing to KS (39). These findings observed alongside the relationship between HHV-8 and MAVS support a model in which genetic and/or virally mediated disruption of RIG-I/MAVS signaling may impair antiviral control of HHV-8 and increase susceptibility to KS.

Despite this strong biologic plausibility, the causal pathway involving *MAVS* genetic variation, HHV-8 infection, and incident KS is unclear. We consider here two hypotheses. First, it is possible that the increased risk of KS is driven by the higher risk of *de novo* HHV-8 infection among those with *MAVS* mutations. HHV-8, like other human herpesviruses, establishes lifelong infection within cellular reservoirs (38). MAVS dysfunction may increase the risk of earlier established HHV-8 infection, thus increasing the risk for KS vis-à-vis time exposed to the virus. Polymorphisms in other immune-modulating genes have been previously associated with HHV-8 infection, but to our knowledge, none have been replicated in independent populations (40).

A second, non-mutually exclusive hypothesis is that *MAVS* genetic variation places patients already infected by HHV-8 infection at higher risk of developing KS. As discussed earlier, HHV-8 gene expression within latently infected cells is influenced by MAVS protein dysfunction, thus risk for KS due to *MAVS* dysfunction may be related to its role in preventing malignant transformation of infected cells.

Although HHV-8 seropositivity is common in Africa, ranging 20–50%, the incidence of KS even at the peak of the HIV pandemic rarely exceeded 30 per 100,000 person-years at risk (3, 7, 41, 42). Thus, while HHV-8 infection is a necessary component cause of KS, it is an insufficient cause on its own. HIV co-infection increases KS risk ~100-fold, yet even in high-prevalence regions, the 5-year cumulative incidence of KS among people living with HIV remains <1%, leaving most with HIV/HHV-8 co-infection without KS (43–45).

In children, KS often develops with normal CD4 counts among those who are already on antiretroviral therapy, possibly related to the effect of HIV infection on the developing immune system (46, 47). In Malawi, for example, pediatric KS occurred after a median of 14 months on therapy with CD4 counts up to 936/µL at diagnosis (46). Moreover, endemic KS arises in otherwise healthy young people without HIV (11, 48). While high HHV-8 seroprevalence likely contributes to the greater burden of KS in Africa, HHV-8 is not absent elsewhere, with prevalence reported as high as 6% in children in the United States (49).

As demonstrated in this study, genetic susceptibility may help explain the unique epidemiology of KS in Africa. Rare familial clusters with mutations in immune-regulatory genes have been reported, and some candidate gene associations are described, though only *HLA-B* and *IL-6* variants (each with <5% frequency in African populations) have been replicated (40, 50–55). In contrast, the *MAVS* variant identified here is common in African populations relative to European populations, ~40% vs. ~15% (31). Its higher frequency may partially account for the greater incidence of KS in Africa, and its functional link to HHV-8 infection further supports its candidacy.

An additional consideration is the apparent difference in genotype–phenotype relationships between *MAVS* rs7269320 SNV and KS in the pediatric and adult cohorts. In children, the association between rs7269320 and KS was most evident under additive and allelic models, whereas in adults, the same variant was associated only under a recessive model with evidence of deviation from additivity. This discrepancy may reflect a combination of biological and methodological factors.

Statistically, additive tests may still detect recessive effects when the minor allele frequency is high, as in the pediatric sample (**Table 1**), whereas the recessive test may have become more powerful in the adult sample with a larger sample size, where homozygote counts were larger, and controls were restricted to HHV-8–seropositive individuals. The pediatric sample was likely underpowered to elucidate the true genotype-phenotype relationship between *MAVS* variability and KS. We may hypothesize that biologically, heterozygosity contributes partially to risk in the context of perinatal HIV infection, ART exposure, and immune maturation in children, while in adults, progression to KS may require homozygous variation in *MAVS*. While the results from the validation adult study are consistent with a non-additive *MAVS* effect on KS risk, further investigation in larger cohorts alongside functional studies will be required to better establish the true genotype-phenotype relationship.

While control populations used in each experiment were similar in composition to cases, direct comparisons of the effect sizes of the rs7269320 - KS association between children and adults should be made cautiously. The effect size within the pediatric discovery experiment (OR = 3.8) was approximately double that of the adult validation experiment (OR = 2.2), a relatively common phenomenon when similar genotype-phenotype relationships are studied between children and adults (16, 56). Importantly there are several differences in the immune risk environment between children and adults that may affect the relative effect of genetic susceptibility to KS, even in similar circumstances of immunosuppression due to HIV. For example, the immune systems of children born with HIV develop within the context of impaired T cell immunity with lifelong effects on immune function (57). Furthermore, adults with HIV infection often have longer cumulative exposure to pro-inflammatory oncogenic viruses, potentially contributing to gene–environment interactions distinct from those in children (58).

We also considered the established epidemiological phenomenon that KS is more common among males than females, particularly in its HIV-negative form (59–61). We did not detect sex-linked genetic associations with KS. Other hypotheses remain, including sex-limited expression of autosomal genetic variation, epigenetic phenomena, and sex-linked environmental exposures. Additionally, the pediatric discovery study was likely underpowered to detect a modest effect size given the X-linked recessive genotype-phenotype relationship.

In light of the relationship between genetic susceptibility and infectious exposures in KS described here, several future directions for research into the unique epidemiology of KS emerge. For example, investigating the relationship between malaria infection, genetic susceptibility, and KS is particularly interesting. The epidemiological maps of *Plasmodium falciparum* and KS overlap across Africa (7, 62). Also, *P. falciparum* endemicity and infection were recently associated with increased prevalence of KSHV seropositivity (63, 64). Furthermore, HHV-8 lytic activity increases in the setting of acute malaria infection (63). An interplay between a relatively high prevalence of *MAVS* variation and *P. falciparum* endemicity may yet explain in part why HIV-negative KS among young people is unique to this region of the world (11).

Several other chronic and recurrent infections common among persons living with HIV contribute to cancer risk and may be further explored with respect to variation in RIG-I/MAVS signaling pathway (58). Epstein-Barr virus (EBV), cytomegalovirus, human papillomavirus, and hepatitis B and C are all mechanistically linked to the RIG-I/MAVS pathway, providing logical next-step avenues for investigation in the context of genetic susceptibility and cancer risk among people living with HIV (65–69). EBV is particularly interesting, given its biological similarities to HHV-8, interface with the RIG-I/MAVS signaling axis, and relationship to endemic Burkitt lymphoma, which also follows the epidemiological map of *P. falciparum* across Africa and is among the most common pediatric cancers in sub-Saharan Africa (69, 70).

Finally, the relationship between genetic variation in the RIG-I/MAVS pathway and classic KS among older adults offers potential for future research, particularly given recent work linking the two (39). We may hypothesize that the risk of KS due to co-factors such as immune senescence and/or drivers of chronic inflammation common among older adults, such as Type II diabetes and obesity, may be potentiated by genetic susceptibility described here (71, 72).

### Strengths and limitations

Despite limited power, this study is strengthened by its discovery-validation design and a patient population spanning both children and adults. To our knowledge, this is the first study in children to identify a risk variant associated with KS, and it is among the few studies in children or adults to replicate its findings in an independent population. This study leveraged the largest pediatric KS cohort in the world, comprising children cared for by the Baylor College of Medicine International Pediatrics AIDS Initiative Network across Africa. The study is further strengthened by the agnostic approach of GWAS (i.e., specific immune-related genes were not targeted *a priori*) and by the identification of a mutation in a gene with a highly plausible, previously described relationship with HHV-8 infection. Finally, this study was unable to elucidate a relationship between *MAVS* variation and KS in children or adults without HIV infection, an important population that is unaffected by improved access to ART and subsequent decreases in HIV-related malignancies. Future multicenter studies will be required to power such an investigation in this relatively rare cancer of young people in the African population.

### Conclusion

In conclusion, we detected a highly biologically plausible relationship between a SNV in the MAVS/RIG-I pathway and risk for HIV-related KS in children and adults. While the findings suggest a possible recessive genotype-phenotype relationship, further study into the relationship between specific alterations to the *MAVS* gene, downstream risk for HHV-8 and/or KS, and ultimately risk for KS itself will be required. If further validated, this finding may be leveraged for improved risk profiling of patients living with HIV with respect to KS, to potentially identify patients at higher risk of relapse, and to be explored as a novel therapeutic target. The role of *MAVS* in other HHV-8-mediated cancers with increased frequency among people living with HIV, particularly Multicentric Castleman Disease and Primary Effusion Lymphoma, should also be explored (73).

## Data Availability

The datasets presented in this article are not readily available because of confidentiality agreement. Requests to access the datasets should be directed to the corresponding author.

## References

[1] Gonçalves PH Uldrick TS Yarchoan R . HIV-associated Kaposi sarcoma and related diseases. Aids. (2017) 31:1903–16. doi: 10.1097/qad.0000000000001567 28609402 PMC6310482

[2] El-Mallawany NK McAtee CL Campbell LR Kazembe PN . Pediatric Kaposi sarcoma in context of the HIV epidemic in sub-Saharan Africa: current perspectives. Pediatr Heal Med Ther. (2018) 9:35–46. doi: 10.2147/phmt.s142816 29722363 PMC5919159

[3] Butler LM Were WA Balinandi S Downing R Dollard S Neilands TB . Human herpesvirus 8 infection in children and adults in a population-based study in rural Uganda. J Infect Dis. (2011) 203:625–34. doi: 10.1093/infdis/jiq092 21273188 PMC3071279

[4] Rees CA Keating EM Lukolyo H Danysh HE Scheurer ME Mehta PS . Mapping the epidemiology of kaposi sarcoma and non‐Hodgkin lymphoma among children in sub‐Saharan africa: A review. Pediatr Blood Cancer. (2016) 63:1325–31. doi: 10.1002/pbc.26021 27082516 PMC7340190

[5] Ward ZJ Yeh JM Bhakta N Frazier AL Girardi F Atun R . Global childhood cancer survival estimates and priority-setting: a simulation-based analysis. Lancet Oncol. (2019) 20:972–83. doi: 10.1016/s1470-2045(19)30273-6 31129029

[6] Haq H Elyanu P Bulsara S Bacha JM Campbell LR El-Mallawany NK . Association between antiretroviral therapy and cancers among children living with HIV in sub-saharan africa. Cancers. (2021) 13:1379. doi: 10.3390/cancers13061379 33803641 PMC8003101

[7] Dollard SC Butler LM Jones AMG Mermin JH Chidzonga M Chipato T . Substantial regional differences in human herpesvirus 8 seroprevalence in sub‐Saharan Africa: Insights on the origin of the “Kaposi’s sarcoma belt. Int J Cancer. (2010) 127:2395–401. doi: 10.1002/ijc.25235 20143397 PMC2895015

[8] Tukei VJ Kekitiinwa A Beasley RP . Prevalence and outcome of HIV-associated Malignancies among children. Aids. (2011) 25:1789–93. doi: 10.1097/qad.0b013e3283498115 21673560

[9] Stefan DC Stones DK Wainwright L Newton R . Kaposi sarcoma in South African children. Pediatr Blood Cancer. (2010) 56:392–6. doi: 10.1002/pbc.22903 21225916

[10] Chagaluka G Stanley C Banda K Depani S Nijram’madzi J Katangwe T . Kaposi’s sarcoma in children: An open randomised trial of vincristine, oral etoposide and a combination of vincristine and bleomycin. Eur J Cancer. (2014) 50:1472–81. doi: 10.1016/j.ejca.2014.02.019 24636877

[11] El-Mallawany NK Villiera J Kamiyango W Peckham-Gregory EC Scheurer ME Allen CE . Endemic Kaposi sarcoma in HIV-negative children and adolescents: an evaluation of overlapping and distinct clinical features in comparison with HIV-related disease. Infect Agents Cancer. (2018) 13:33. doi: 10.1186/s13027-018-0207-4 30455728 PMC6230225

[12] Anderson CA Pettersson FH Clarke GM Cardon LR Morris AP Zondervan KT . Data quality control in genetic case-control association studies. Nat Protoc. (2010) 5:1564–73. doi: 10.1038/nprot.2010.116 21085122 PMC3025522

[13] Gao F Chang D Biddanda A Ma L Guo Y Zhou Z . XWAS: A software toolset for genetic data analysis and association studies of the X chromosome. J Hered. (2015) 106:666–71. doi: 10.1093/jhered/esv059 26268243 PMC4567842

[14] Price AL Patterson NJ Plenge RM Weinblatt ME Shadick NA Reich D . Principal components analysis corrects for stratification in genome-wide association studies. Nat Genet. (2006) 38:904–9. doi: 10.1038/ng1847 16862161

[15] Archer NP Perez‐Andreu V Scheurer ME Rabin KR Peckham‐Gregory EC Plon SE . Family‐based exome‐wide assessment of maternal genetic effects on susceptibility to childhood B‐cell acute lymphoblastic leukemia in hispanics. Cancer. (2016) 122:3697–704. doi: 10.1002/cncr.30241 27529658 PMC5115939

[16] Peckham-Gregory EC Chakraborty R Scheurer ME Belmont JW Abhyankar H Sengal AG . A genome-wide association study of LCH identifies a variant in SMAD6 associated with susceptibility. Blood. (2017) 130:2229–32. doi: 10.1182/blood-2017-08-800565 28935696 PMC5691246

[17] Clarke GM Anderson CA Pettersson FH Cardon LR Morris AP Zondervan KT . Basic statistical analysis in genetic case-control studies. Nat Protoc. (2011) 6:121–33. doi: 10.1038/nprot.2010.182 21293453 PMC3154648

[18] Chang D Gao F Slavney A Ma L Waldman YY Sams AJ . Accounting for eXentricities: analysis of the X chromosome in GWAS reveals X-linked genes implicated in autoimmune diseases. PloS One. (2014) 9:e113684. doi: 10.1371/journal.pone.0113684 25479423 PMC4257614

[19] Ward LD Kellis M . HaploReg: a resource for exploring chromatin states, conservation, and regulatory motif alterations within sets of genetically linked variants. Nucleic Acids Res. (2011) 40:D930–4. doi: 10.1093/nar/gkr917 22064851 PMC3245002

[20] Flanagan SE Patch A-M Ellard S . Using SIFT and polyPhen to predict loss-of-function and gain-of-function mutations. Genet Test Mol Bioma. (2010) 14:533–7. doi: 10.1089/gtmb.2010.0036 20642364

[21] Purcell S Neale B Todd-Brown K Thomas L Ferreira MAR Bender D . PLINK: A tool set for whole-genome association and population-based linkage analyses. Am J Hum Genet. (2007) 81:559–75. doi: 10.1086/519795 17701901 PMC1950838

[22] Team RC . R: A Language and Environment for Statistical Computing (2025). Available online at: https://www.R-project.org/ (Accessed January 1, 2025).

[23] Byakwaga H Hunt PW Laker-Oketta M Glidden DV Huang Y Bwana BM . The kynurenine pathway of tryptophan catabolism and AIDS-associated kaposi sarcoma in africa. JAIDS J Acquir Immune Defic Syndr. (2015) 70:296–303. doi: 10.1097/qai.0000000000000747 26181812 PMC4607630

[24] Scott L Laker-Oketta M Byakwaga H Glidden D Mwebesa B Muzoora C . Impact of kaposi sarcoma on quality of life amongst HIV-infected adults initiating antiretroviral therapy in east africa. medRxiv. (2023). doi: 10.1101/2023.07.21.23292658 37546765 PMC10402209

[25] Haberer JE Kiwanuka J Nansera D Muzoora C Hunt PW So J . Realtime adherence monitoring of antiretroviral therapy among hiv-infected adults and children in rural Uganda. AIDS. (2013) 27:2166–8. doi: 10.1097/qad.0b013e328363b53f 23751260 PMC3868644

[26] Lee GQ Bangsberg DR Muzoora C Boum Y Oyugi JH Emenyonu N . Prevalence and virologic consequences of transmitted HIV-1 drug resistance in Uganda. AIDS Res Hum Retroviruses. (2014) 30:896–906. doi: 10.1089/aid.2014.0043 24960249 PMC4151058

[27] McCluskey SM Lee GQ Kamelian K Kembabazi A Musinguzi N Bwana MB . Increasing prevalence of HIV pretreatment drug resistance in women but not men in rural Uganda during 2005–2013. AIDS Patient Care STDs. (2018) 32:257–64. doi: 10.1089/apc.2018.0020 29985647 PMC6034395

[28] West JA Wicks M Gregory SM Chugh P Jacobs SR Zhang Z . An important role for mitochondrial antiviral signaling protein in the kaposi’s sarcoma-associated herpesvirus life cycle. J Virol. (2014) 88:5778–87. doi: 10.1128/jvi.03226-13 24623417 PMC4019080

[29] Hwang KY Choi YB . Modulation of mitochondrial antiviral signaling by human herpesvirus 8 interferon regulatory factor 1. J Virol. (2015) 90:506–20. doi: 10.1128/jvi.01903-15 26512076 PMC4702585

[30] Auton A Abecasis GR Altshuler DM Durbin RM Abecasis GR Bentley DR . A global reference for human genetic variation. Nature. (2015) 526:68–74. doi: 10.1038/nature15393 26432245 PMC4750478

[31] Sherry ST Ward M-H Kholodov M Baker J Phan L Smigielski EM . dbSNP: the NCBI database of genetic variation. Nucleic Acids Res. (2001) 29:308–11. doi: 10.1093/nar/29.1.308 11125122 PMC29783

[32] Pothlichet J Niewold TB Vitour D Solhonne B Crow MK Si‐Tahar M . A loss‐of‐function variant of the antiviral molecule MAVS is associated with a subset of systemic lupus patients. EMBO Mol Med. (2011) 3:142–52. doi: 10.1002/emmm.201000120 21268286 PMC3395111

[33] Xing F Matsumiya T Hayakari R Yoshida H Kawaguchi S Takahashi I . Alteration of antiviral signalling by single nucleotide polymorphisms (SNPs) of mitochondrial antiviral signalling protein (MAVS). PloS One. (2016) 11:e0151173. doi: 10.1371/journal.pone.0151173 26954674 PMC4783065

[34] Choi YB Choi Y Harhaj EW . Peroxisomes support human herpesvirus 8 latency by stabilizing the viral oncogenic protein vFLIP via the MAVS-TRAF complex. PloS Pathog. (2018) 14:e1007058. doi: 10.1371/journal.ppat.1007058 29746593 PMC5963799

[35] Seth RB Sun L Ea C-K Chen ZJ . Identification and characterization of MAVS, a mitochondrial antiviral signaling protein that activates NF-κB and IRF3. Cell. (2005) 122:669–82. doi: 10.1016/j.cell.2005.08.012 16125763

[36] Yu K Tian H Deng H . PPM1G restricts innate immune signaling mediated by STING and MAVS and is hijacked by KSHV for immune evasion. Sci Adv. (2020) 6:eabd0276. doi: 10.1126/sciadv.abd0276 33219031 PMC7679160

[37] Zhu Q McElroy R Machhar JS Cassel J Zheng Z Mansoori B . Kaposi’s sarcoma-associated herpesvirus induces mitochondrial fission to evade host immune responses and promote viral production. Nat Microbiol. (2025) 10:1501–20. doi: 10.1038/s41564-025-02018-3 40404827 PMC12337130

[38] Dittmer DP Damania B . Kaposi sarcoma–associated herpesvirus: immunobiology, oncogenesis, and therapy. J Clin Invest. (2016) 126:3165–75. doi: 10.1172/jci84418 27584730 PMC5004954

[39] Roussel L Bernier S Langelier M Sun Y Mak B Li Y . Human retinoic acid–inducible gene I deficiency is associated with susceptibility to classic Kaposi sarcoma. J Allergy Clin Immunol. (2026). doi: 10.1016/j.jaci.2026.02.027 41780571

[40] Blumenthal MJ Castro EMC Whitby D Katz AA Schäfer G . Evidence for altered host genetic factors in KSHV infection and KSHV‐related disease development. Rev Med Virol. (2021) 31:e2160. doi: 10.1002/rmv.2160 33043529 PMC8047912

[41] Parkin DM Sitas F Chirenje M Stein L Abratt R Wabinga H . Part I: Cancer in Indigenous Africans—burden, distribution, and trends. Lancet Oncol. (2008) 9:683–92. doi: 10.1016/s1470-2045(08)70175-x 18598933

[42] Rohner E Wyss N Trelle S Mbulaiteye SM Egger M Novak U . HHV-8 seroprevalence: a global view. Syst Rev. (2014) 3:11. doi: 10.1186/2046-4053-3-11 24521144 PMC3925012

[43] Rohner E Valeri F Maskew M Prozesky H Rabie H Garone D . Incidence rate of kaposi sarcoma in HIV-infected patients on antiretroviral therapy in southern africa. Jaids J Acquir Immune Defic Syndromes. (2014) 67:547–54. doi: 10.1097/qai.0000000000000360 25393941 PMC4231535

[44] Stolka K Ndom P Hemingway-Foday J Iriondo-Perez J Miley W Labo N . Risk factors for Kaposi’s sarcoma among HIV-positive individuals in a case control study in Cameroon. Cancer Epidemiol. (2014) 38:137–43. doi: 10.1016/j.canep.2014.02.006 24631417 PMC4075442

[45] Motlhale M Muchengeti M Bradshaw D Chen WC Singini MG Villiers C . Kaposi sarcoma‐associated herpesvirus, HIV‐1 and Kaposi sarcoma risk in black South Africans diagnosed with cancer during antiretroviral treatment rollout. Int J Cancer. (2023) 152:2081–9. doi: 10.1002/ijc.34454 36727526

[46] Silverstein A Kamiyango W Villiera J Peckham‐Gregory EC McAtee CL Scheurer ME . Long‐term outcomes for children and adolescents with Kaposi sarcoma. HIV Med. (2022) 23:197–203. doi: 10.1111/hiv.13191 34634187 PMC9121366

[47] Caro-Vegas C Peng A Juarez A Silverstein A Kamiyango W Villiera J . Pediatric HIV+ Kaposi sarcoma exhibits clinical, virological, and molecular features different from the adult disease. JCI Insight. (2023) 8:e167854. doi: 10.1172/jci.insight.167854 37991023 PMC10721314

[48] Friedman-Kien AE Saltzman BR . Clinical manifestations of classical, endemic African, and epidemic AIDS-associated Kaposi’s sarcoma. J Am Acad Dermatol. (1990) 22:1237–50. doi: 10.1016/0190-9622(90)70169-i 2193952

[49] Anderson LA Li Y Graubard BI Whitby D Mbisa G Tan S . Human herpesvirus 8 seroprevalence among children and adolescents in the United States. Pediatr Infect Dis J. (2008) 27:661–4. doi: 10.1097/inf.0b013e3181691740 18536622

[50] Foster CB Lehrnbecher T Samuels S Stein S Mol F Metcalf JA . An IL6 promoter polymorphism is associated with a lifetime risk of development of Kaposi sarcoma in men infected with human immunodeficiency virus. Blood. (2000) 96:2562–7. doi: 10.1182/blood.v96.7.2562.h8002562_2562_2567 11001912

[51] Williams F Meenagh A Darke C Acosta A Daar AS Gorodezky C . Analysis of the distribution of HLA-B alleles in populations from five continents. Hum Immunol. (2001) 62:645–50. doi: 10.1016/s0198-8859(01)00247-6 11390040

[52] Guttman‐Yassky E Cohen A Kra‐Oz Z Friedman‐Birnbaum R Sprecher E Zaltzman N . Familial clustering of classic Kaposi sarcoma. J Infect Dis. (2004) 189:2023–6. doi: 10.1086/386308 15143469

[53] Byun M Abhyankar A Lelarge V Plancoulaine S Palanduz A Telhan L . Whole-exome sequencing-based discovery of STIM1 deficiency in a child with fatal classic Kaposi sarcoma. J Exp Med. (2010) 207:2307–12. doi: 10.1084/jem.20101597 20876309 PMC2964585

[54] Byun M Ma CS Akçay A Pedergnana V Palendira U Myoung J . Inherited human OX40 deficiency underlying classic Kaposi sarcoma of childhood. J Exp Med. (2013) 210:1743–59. doi: 10.1084/jem.20130592 23897980 PMC3754857

[55] Aissani B Boehme AK Wiener HW Shrestha S Jacobson LP Kaslow RA . SNP screening of central MHC-identified HLA-DMB as a candidate susceptibility gene for HIV-related Kaposi’s sarcoma. Genes Immun. (2014) 15:424–9. doi: 10.1038/gene.2014.42 25008864 PMC4174341

[56] Agopian AJ Eastcott LM Mitchell LE . Age of onset and effect size in genome‐wide association studies. Birth Defects Res Part Clin Mol Teratol. (2012) 94:908–11. doi: 10.1002/bdra.23066 22933422 PMC4219508

[57] Muenchhoff M Prendergast AJ Goulder PJR . Immunity to HIV in early life. Front Immunol. (2014) 5:391. doi: 10.3389/fimmu.2014.00391 25161656 PMC4130105

[58] Plummer M Martel C Vignat J Ferlay J Bray F Franceschi S . Global burden of cancers attributable to infections in 2012: a synthetic analysis. Lancet Glob Heal. (2016) 4:e609–16. doi: 10.1016/s2214-109x(16)30143-7 27470177

[59] Dutz W Stout AP . Kaposi’s sarcoma in infants and children. Cancer. (1960) 13:684–93. doi: 10.1002/1097-0142(196007/08)13:4<684::aid-cncr2820130408>3.0.co;2-g 13818924

[60] Cox CM El‐Mallawany NK Kabue M Kovarik C Schutze GE Kazembe PN . Clinical characteristics and outcomes of HIV‐infected children diagnosed with kaposi sarcoma in Malawi and Botswana. Pediatr Blood Cancer. (2013) 60:1274–80. doi: 10.1002/pbc.24516 23487320

[61] Macken M Dale H Moyo D Chakmata E Depani S Israels T . Triple therapy of vincristine, bleomycin and etoposide for children with Kaposi sarcoma: Results of a study in Malawian children. Pediatr Blood Cancer. (2017) 65:e26841. doi: 10.1002/pbc.26841 28988435

[62] Gething PW Casey DC Weiss DJ Bisanzio D Bhatt S Cameron E . Mapping Plasmodium falciparum mortality in Africa between 1990 and 2015. N Engl J Med. (2016) 375:2435–45. doi: 10.1056/nejmoa1606701 27723434 PMC5484406

[63] Oluoch PO Forconi CS Oduor CI Ritacco DA Akala HM Bailey JA . Distinctive Kaposi sarcoma-associated herpesvirus serological profile during acute Plasmodium falciparum malaria episodes. Int J Mol Sci. (2023) 24:6711. doi: 10.3390/ijms24076711 37047683 PMC10095526

[64] Nalwoga A Sabourin KR Miley W Jackson C Maktabi M Labo N . Plasmodium falciparum malaria is associated with increased Kaposi sarcoma–associated herpesvirus (KSHV) seropositivity and higher KSHV antibody breadth and magnitude: Results of a case-control study from rural Uganda. J Infect Dis. (2023) 229:432–42. doi: 10.1093/infdis/jiad308 37536670 PMC10873168

[65] Meylan E Curran J Hofmann K Moradpour D Binder M Bartenschlager R . Cardif is an adaptor protein in the RIG-I antiviral pathway and is targeted by hepatitis C virus. Nature. (2005) 437:1167–72. doi: 10.1038/nature04193 16177806

[66] Scott I . Degradation of RIG-I following cytomegalovirus infection is independent of apoptosis. Microbes Infect. (2009) 11:973–9. doi: 10.1016/j.micinf.2009.07.001 19591957 PMC2741008

[67] Wei C Ni C Song T Liu Y Yang X Zheng Z . The hepatitis B virus X protein disrupts innate immunity by downregulating mitochondrial antiviral signaling protein. J Immunol. (2010) 185:1158–68. doi: 10.4049/jimmunol.0903874 20554965

[68] Chiang C Pauli E-K Biryukov J Feister KF Meng M White EA . The human papillomavirus E6 oncoprotein targets USP15 and TRIM25 to suppress RIG-I-mediated innate immune signaling. J Virol. (2018) 92:e01737–17. doi: 10.1128/jvi.01737-17 29263274 PMC5827370

[69] Lui W-Y Bharti A Wong N-H Jangra S Botelho MG Yuen K-S . Suppression of cGAS- and RIG-I-mediated innate immune signaling by Epstein-Barr virus deubiquitinase BPLF1. PloS Pathog. (2023) 19:e1011186. doi: 10.1371/journal.ppat.1011186 36802409 PMC9983872

[70] Molyneux EM Rochford R Griffin B Newton R Jackson G Menon G . Burkitt’s lymphoma. Lancet. (2012) 379:1234–44. doi: 10.1016/s0140-6736(11)61177-x 22333947

[71] Greten FR Grivennikov SI . Inflammation and cancer: Triggers, mechanisms, and consequences. Immunity. (2019) 51:27–41. doi: 10.1016/j.immuni.2019.06.025 31315034 PMC6831096

[72] Royston L Jary A Berini CA Mabanga T Lin J Pagliuzza A . Similar viral and immune characteristics of Kaposi sarcoma in ART-treated people living with HIV and older patients with classic Kaposi sarcoma. Open Forum Infect Dis. (2024) 11:ofae404. doi: 10.1093/ofid/ofae404 39100526 PMC11295207

[73] Polizzotto MN Uldrick TS Wyvill KM Aleman K Marshall V Wang V . Clinical features and outcomes of patients with symptomatic Kaposi sarcoma herpesvirus (KSHV)-associated inflammation: Prospective characterization of KSHV inflammatory cytokine syndrome (KICS). Clin Infect Dis. (2016) 62:730–8. doi: 10.1093/cid/civ996 26658701 PMC4772848

