# Supplementary material for: Genome-wide association study identifies and validates genetic variation in the RIG-I/MAVS signaling pathway associated with HIV-related Kaposi sarcoma in children and adults
Source: Front Oncol. 2026 Jun 29;16:1796311. doi: 10.3389/fonc.2026.1796311 (PMC13357122; doi:10.3389/fonc.2026.1796311)
Supplement: Supplementary file 1 [file DataSheet1.docx]

**SUPPLEMENTAL MATERIALS - *Genome-wide association study identifies and validates genetic variation in the RIG-I/MAVS signaling pathway associated with HIV-related Kaposi sarcoma in children and adults***

**I. Supplemental analysis 1: Control by principal components**

The allelic association test used in the pediatric discovery experiment is a 1-degree-of-freedom 2x2 chi-square test that does not accommodate covariate adjustment, including principal components. When the log-additive logistic regression model in the pediatric discovery experiment was adjusted for a first principal component, the association with rs7269320 was preserved (OR = 4.9; p = 6.8×10^-6^; λ λ = 0.96). Similarly, when the recessive logistic regression model used in the adult validation experiment was adjusted for the first principal component, the association remained nearly identical (OR = 2.1; p = 0.04; λ = 0.8), albeit with attenuated statistical significance.

**II. Supplemental analysis 2: Age and sex adjustment of primary pediatric and adult models**

Neither the pediatric nor the adult models showed substantial changes in the association between rs7269320 and *MAVS* when adjusted for age or sex.

| **MODEL** | **RESULT (*rs7269320 association*)** |
| --- | --- |
| Pediatric (discovery) log-additive logistic regression model |  |
| rs7269320 | OR = 4.9; p = 5.4×10^-6^ |
| rs7269320 + sex | OR = 8.7; p = 2.6×10^-6^ |
| rs7269320 + age | OR = 7.4; p = 1.9×10^-5^ |
| Adult (validation) recessive logistic regression model |  |
| rs7269320 | OR = 2.2; p = 0.006 |
| rs7269320 + sex | OR = 2.2; p = 0.009 |
| rs7269320 + age | OR = 2.2; p = 0.006 |

**II. Supplemental Table**

**Single nucleotide variants reaching suspected significance threshold in pediatric genome wide association study discovery set, allelic χ^2^ test**

| SNV | Alleles | Gene | Functional consequence | Name | P-value | Odds ratio |
| --- | --- | --- | --- | --- | --- | --- |
| rs9411461 | G>A | *GBGT1* | Upstream variant | Globoside alpha-1,3-N-acetylgalactosaminyltransferase 1 | 8.5×10^-7^ | 0.2 |
| rs7269320 | C>T | *MAVS* | Missense | Mitochondrial antiviral signaling protein | 9.7×10^-7^ | 3.8 |
| rs7994909 | T>C | *Intergenic* | None described |  | 3.9×10^-6^ | 5.0 |
| rs12465484 | T>A | *TCF7L1* | Intron variant | Transcription factor 7-like 1 | 4.3×10^-6^ | 8.2 |
| rs355849 | T>C | *COBLL1* | Intron variant | Cordon-Bleu WH2 Repeat Protein Like 1 | 5.0×10^-6^ | 5.6 |
| rs73027947 | G>A | *FCER1A* | Intron variant | Fc Epsilon Receptor Ia | 5.3×10^-6^ | 6.2 |
| rs938818 | G>A,C,T | *ARHGAP15* | Intron variant | Rho GTPase-activating protein 15 | 7.7×10^-6^ | 0.3 |
| rs1673398 | A>G | *PKHD1L1* | Synonymous variant | Polycystic Kidney and Hepatic Disease 1-Like 1 | 8.5×10^-6^ | 0.2 |

**III. Supplemental Figure 1. Q-Q plot of pediatric allelic association test
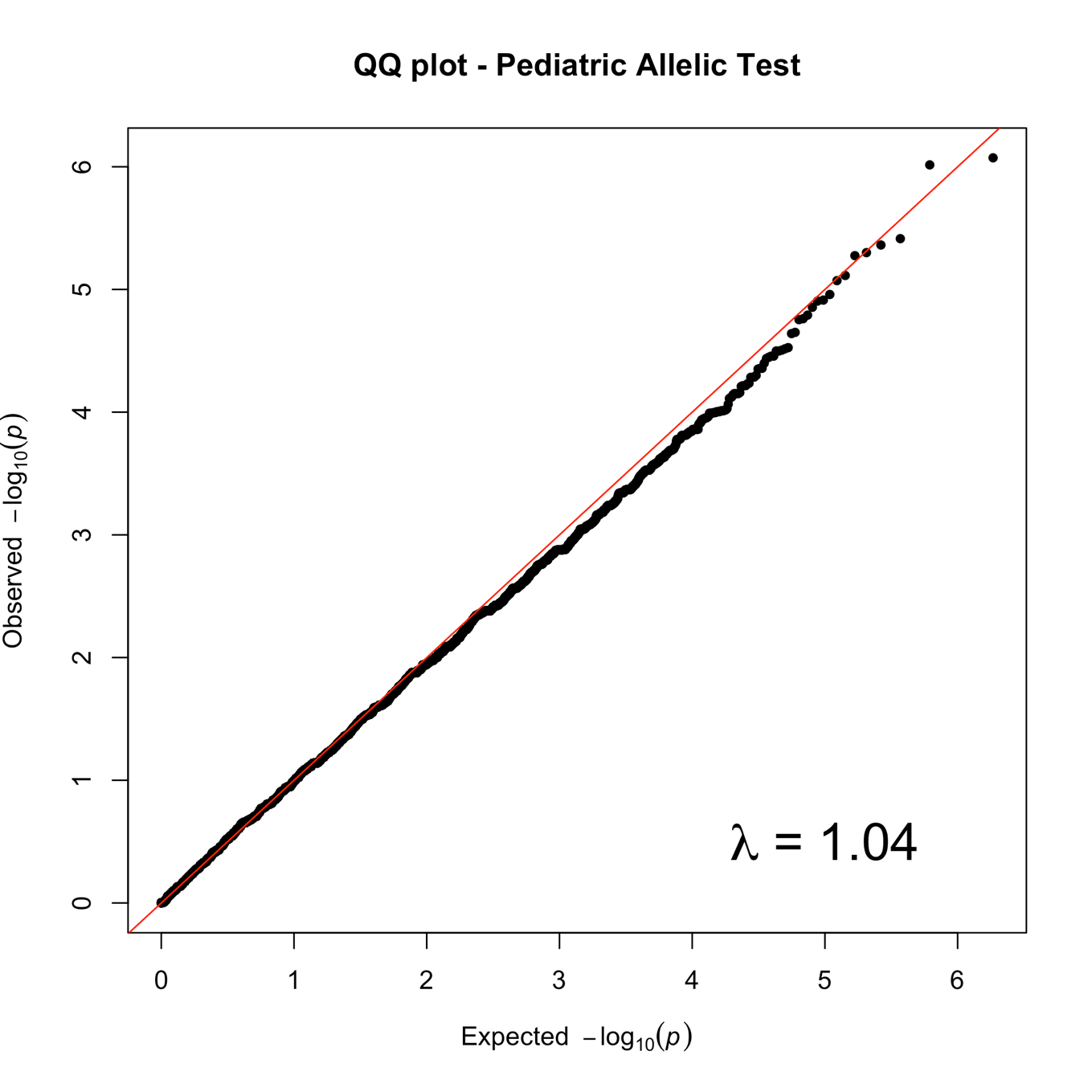
**

**IV. Supplemental Figure 2. Q-Q plot, pediatric log-additive test
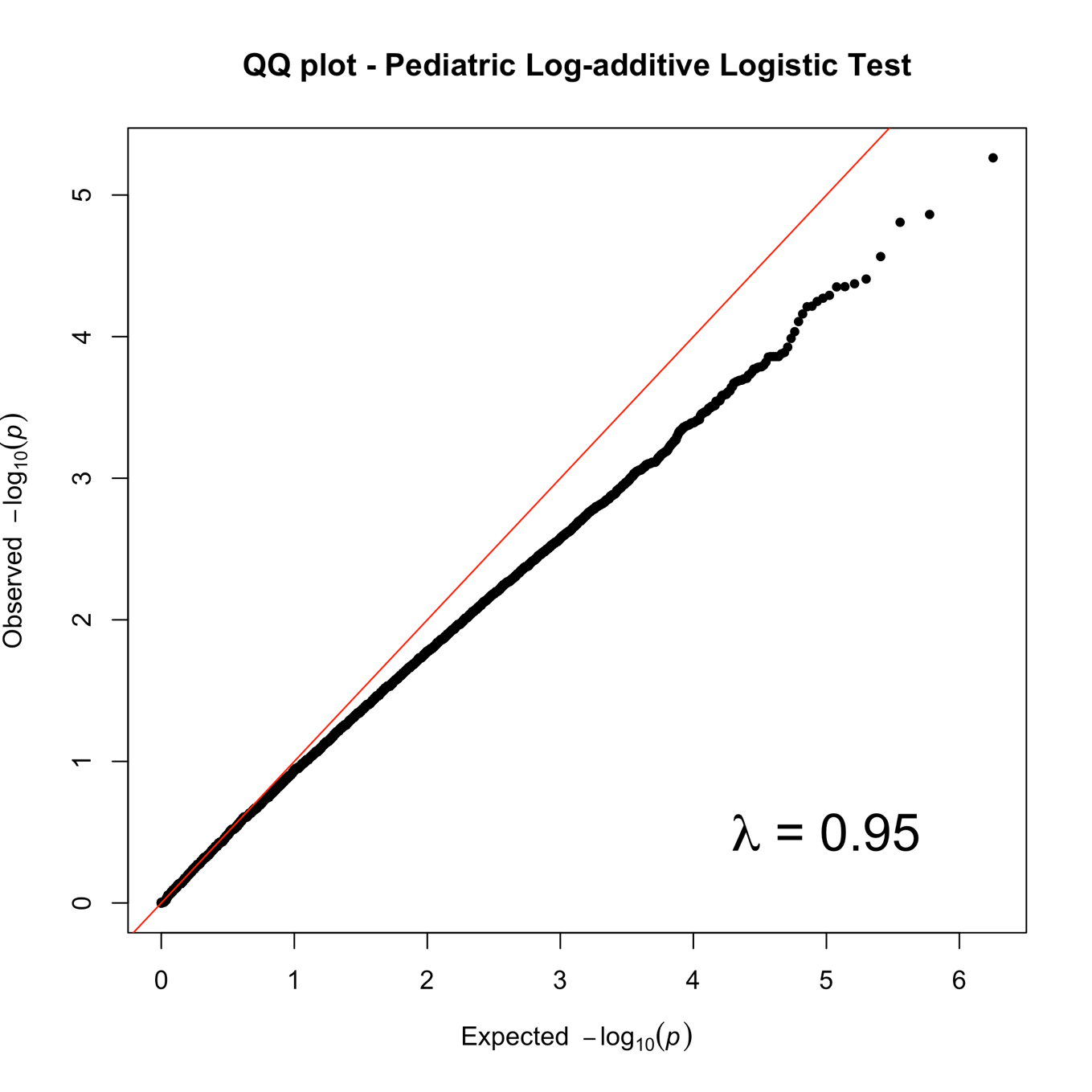
**

**V. Supplemental Figure 3. Q-Q plot of the adult recessive logistic regression model
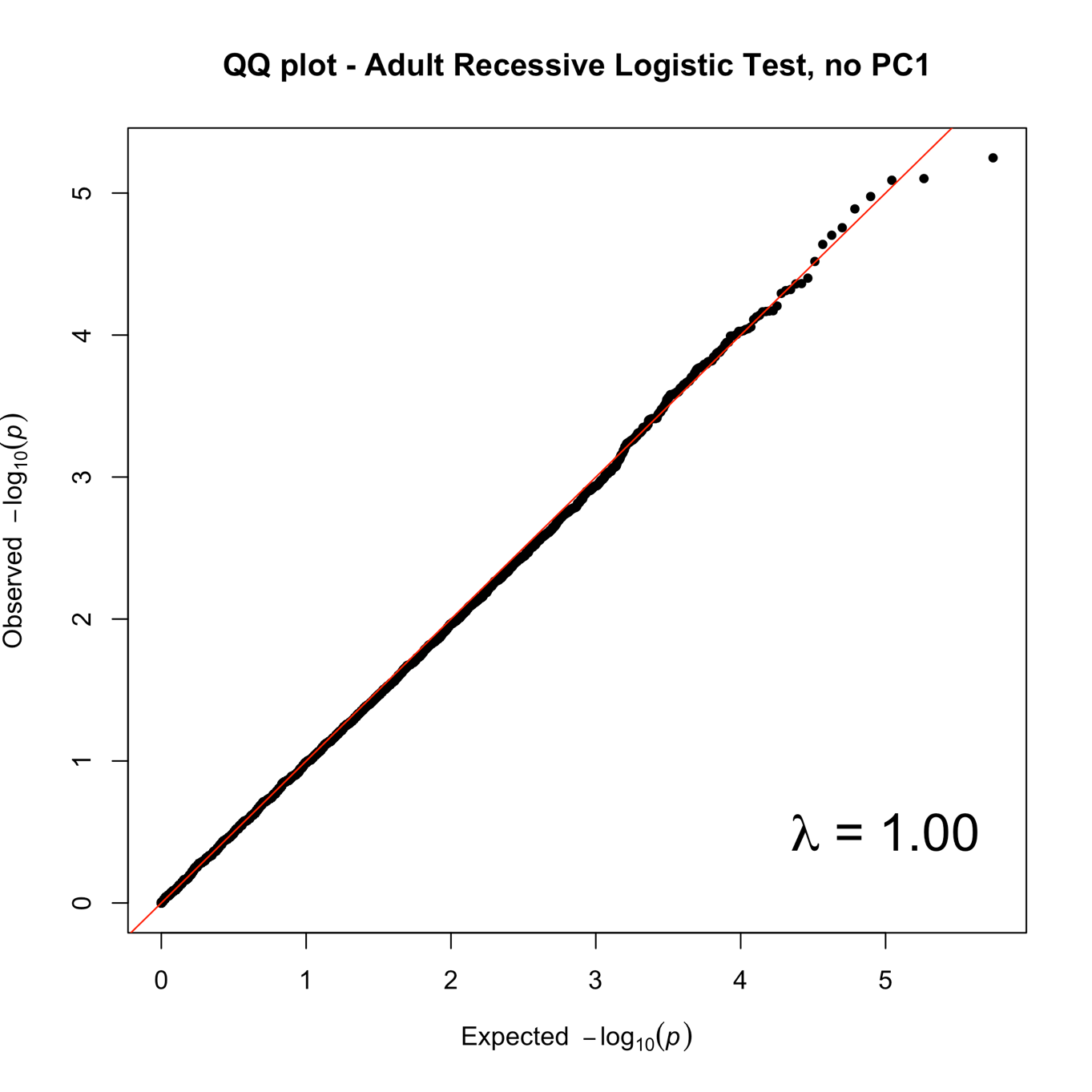
**
